# PW02-021 - SAA1 is the strongest predictor of AA in TRAPS

**DOI:** 10.1186/1546-0096-11-S1-A161

**Published:** 2013-11-08

**Authors:** L Obici, HJ Lachmann, I Touitou, G Palladini, S Donadei, M Pasotti, L Cantarini, K Gerhold, A Meini, N Toplak, P Woo, A Martini, G Merlini, M Gattorno

**Affiliations:** 1 Amyloid Research and Treatment Centre, IRCCS Fondazione Policlinico San Matteo, Pavia, Italy; 2 National Amyloidosis Centre, University College London Medical School, London, UK; 3 INSERM U844, CHRU Montpellier, Montpellier, France; 4 Laboratori Analisi Chimico Cliniche, IRCCS Fondazione Policlinico San Matteo, Pavia, Italy; 5 Rheumatology Unit, Policlinico Le Scotte, Siena, Italy; 6 Department of Pediatric Rheumatology, Charité Hospital, Berlin, Germany; 7 Pediatric Immunology and Rheumatology, Spedali Civili, Brescia, Italy; 8 University Children's Hospital, University Medical Centre Ljubljana, Ljubljana, Slovenia; 9 Centre of Pediatric and Adolescent Rheumatology, UCL, London, UK; 10 Giannina Gaslini Institute, Genova, Italy

## Introduction

AA amyloidosis is the most severe complication of hereditary autoinflammatory diseases. In TRAPS it has been reported to occur in approximately 25% of patients in the absence of an effective treatment. However, susceptibility to AA is difficult to predict. Identification of key risk factors affecting the development of AA would improve the clinical management of TRAPS patients, by allowing a more tailored treatment approach.

## Objectives

To evaluate the relative contribution of clinical and genetic factors to the risk of AA amyloidosis in TRAPS.

## Methods

Clinical data were obtained from the EUROFEVER/EUROTRAPS web-based registry. Inclusion criteria for this study were: age ≥ 18 years at last follow-up, identification of a *TNFRSF1A* mutation and written informed consent. DNA was available for patients recruited into the EUROTRAPS research project and *SAA1* was genotyped by direct sequencing of exon 3.

## Results

104 patients (51 males, 49%) with TRAPS (39 different mutations) were included in the study. Median age was 41 years (range 18-88), median age at TRAPS onset was 6 years (range 0.5-63), and median age at diagnosis was 37 years (10-81). 21 patients had AA amyloidosis, with a median age at AA onset of 38 years (range 18-76). Family history for AA amyloidosis was observed in 33 patients (32%). *SAA1* genotype was established in 89/104 patients and 31 (35%) were homozygous for the *SAA1.1* allele. 77 patients (74%) had a clearly pathogenic *TNFRSF1A* variant. 27 had either P46L or R92Q.

At univariate analysis, family history for amyloidosis, *SAA1.1* homozygosity, disease course, age at TRAPS onset and the type of mutation were significantly associated with AA amyloidosis. At multivariate analysis homozygosity for *SAA1.1* and age at TRAPS onset independently predicted development of renal amyloidosis. *SAA1.1/1.1* genotype was the variable with the strongest influence on AA development, with a 5.3 fold increased risk whereas older age at TRAPS onset was associated with a reduced risk of AA amyloidosis. Survival according to SAA1 genotype (*SAA1.1/SAA1.1* versus all other genotypes) was estimated by Kaplan-Meier analysis. Median amyloid free survival from birth was 47 years vs. not reached (p=0.01).

## Conclusion

Homozygosity for the *SAA1.1* allele is the strongest predictor of AA risk in TRAPS. This result is extremely relevant for the clinical management of TRAPS patients and supports *SAA1* genotyping on a routine basis to guide treatment approach.

## Disclosure of interest

None declared.

